# One of the Largest Reported Ciliated Foregut Cysts of the Gallbladder Mimicking a Gallbladder Diverticulum

**DOI:** 10.7759/cureus.106371

**Published:** 2026-04-03

**Authors:** Yanina Nikolaus

**Affiliations:** 1 Pathology, Marshall University Joan C. Edwards School of Medicine, Huntington, USA

**Keywords:** ciliated foregut cyst, congenital foregut anomaly, gallbladder cyst, gallbladder diverticulum mimic, hepatobiliary pathology

## Abstract

Ciliated foregut cysts are rare congenital lesions derived from abnormal budding of the primitive foregut and are most commonly identified in the mediastinum or liver, where they may also be referred to as bronchogenic-type cysts. Occurrence within the gallbladder is extremely uncommon, with only a limited number of cases reported in the literature.

We present the case of a 29-year-old male who presented with right upper quadrant abdominal pain, nausea, and vomiting. Computed tomography and ultrasound revealed a cystic lesion adjacent to the gallbladder that was initially interpreted radiologically as a probable gallbladder diverticulum. The patient subsequently underwent laparoscopic cholecystectomy. Gross examination demonstrated a 3.5 cm subserosal cystic lesion within the gallbladder wall. Histologic evaluation revealed a cyst lined by pseudostratified ciliated columnar (respiratory-type) epithelium with mucinous differentiation, consistent with a ciliated foregut cyst. No dysplasia or malignancy was identified. The patient’s postoperative course was uncomplicated.

Ciliated foregut cysts of the gallbladder may mimic gallbladder diverticula or other cystic lesions on imaging, making preoperative diagnosis challenging. The cyst in the present case measured 3.5 cm and represents one of the largest reported ciliated foregut cysts of the gallbladder in the literature. This case highlights the importance of histopathologic evaluation and the need to consider a ciliated foregut cyst in the differential diagnosis of cystic lesions associated with the gallbladder.

## Introduction

Ciliated foregut cysts are rare congenital lesions that arise from abnormal budding or sequestration of the primitive foregut during embryologic development. These cysts are most commonly identified in the mediastinum and liver, where they may also be referred to as bronchogenic-type cysts, but they have only rarely been described in the gallbladder [1-3]. Histologically, these cysts are characterized by a lining of pseudostratified ciliated columnar (respiratory-type) epithelium, often with mucin-secreting cells and occasionally a smooth muscle layer within the cyst wall [1-3].

Cystic lesions associated with the gallbladder are uncommon and include gallbladder diverticula, duplication cysts, choledochal cysts, and mucinous cystic neoplasms. Among these, gallbladder diverticula are important radiologic mimics because they may appear as cystic structures adjacent to the gallbladder and can be difficult to distinguish from other cystic lesions on imaging studies [4-6]. As a result, preoperative diagnosis of ciliated foregut cysts of the gallbladder is challenging and is most often established only after histopathologic examination.

Due to their rarity and nonspecific imaging findings, ciliated foregut cysts of the gallbladder may be misinterpreted as more common gallbladder abnormalities, particularly gallbladder diverticula. Recognition of this entity is important for both clinicians and pathologists, as definitive diagnosis relies on histologic identification of pseudostratified ciliated respiratory-type epithelium [1-3,7-10].

We present this case due to the large size of the cyst and its radiologic appearance mimicking a gallbladder diverticulum, representing an important diagnostic pitfall and one of the largest reported ciliated foregut cysts of the gallbladder in the literature.

## Case presentation

This case occurred in early 2026 at a community hospital in the United States. A 29-year-old male presented to the emergency department with a several-day history of right upper quadrant abdominal pain associated with nausea and vomiting. The patient reported decreased oral intake due to persistent nausea but denied fever, chills, or changes in bowel habits. His past medical history was notable for hypertension, and he had no prior abdominal surgeries.

**Figure 1:**
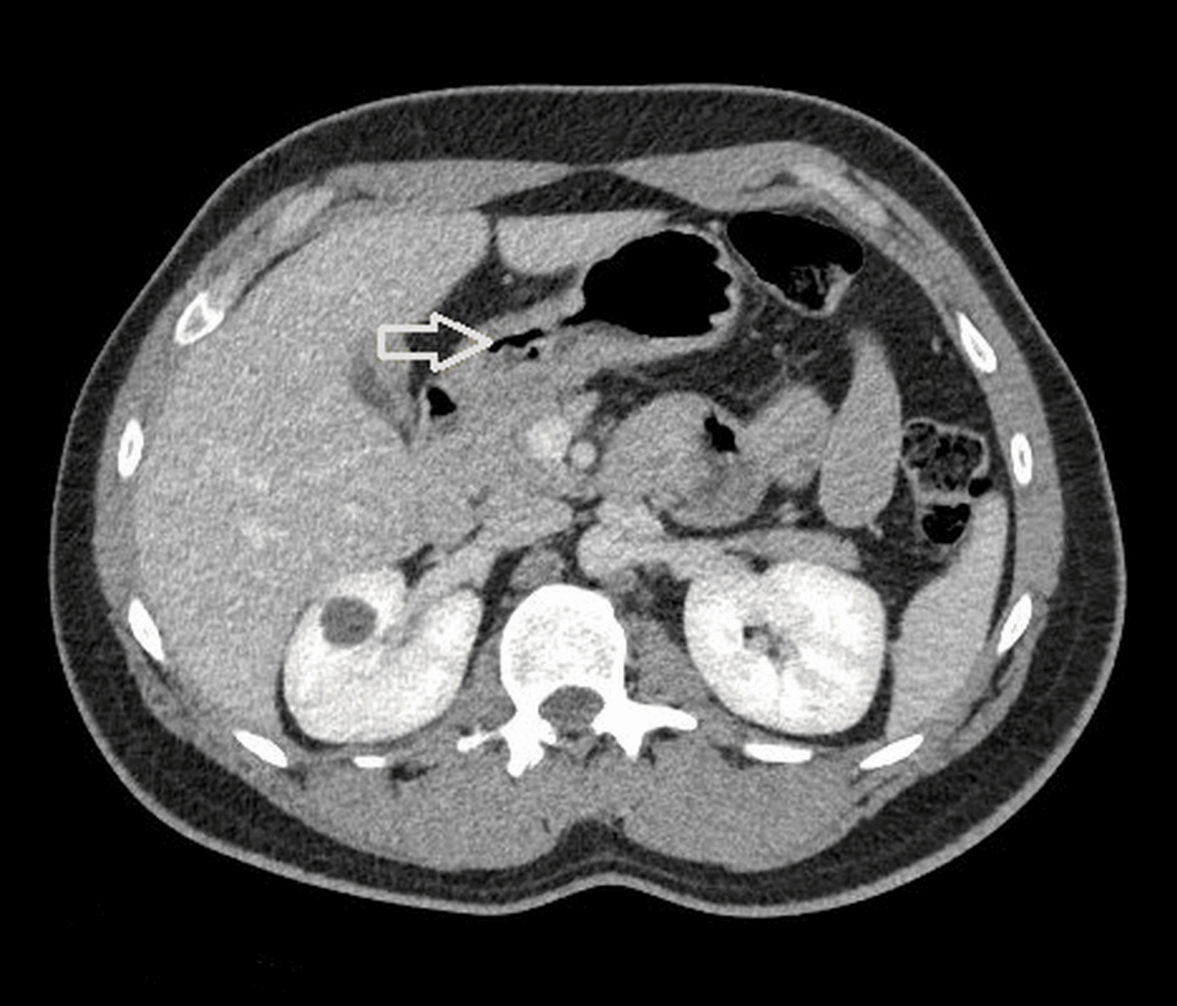
Axial contrast-enhanced CT of the abdomen demonstrating a well-circumscribed low-attenuation cystic lesion adjacent to the gallbladder (arrow), initially interpreted radiologically as a probable gallbladder diverticulum

Computed tomography (CT) of the abdomen and pelvis revealed an indeterminate 1.5 cm low-attenuation nodular lesion located anterior to the gallbladder (Figure 1). No other acute intra-abdominal abnormalities were identified. A follow-up right upper quadrant ultrasound demonstrated a 2.0 cm anechoic structure abutting the gallbladder, which was interpreted radiologically as a probable gallbladder diverticulum. Due to persistent symptoms and subsequent development of acute cholecystitis, the patient was admitted for further management and underwent laparoscopic cholecystectomy.

**Figure 2:**
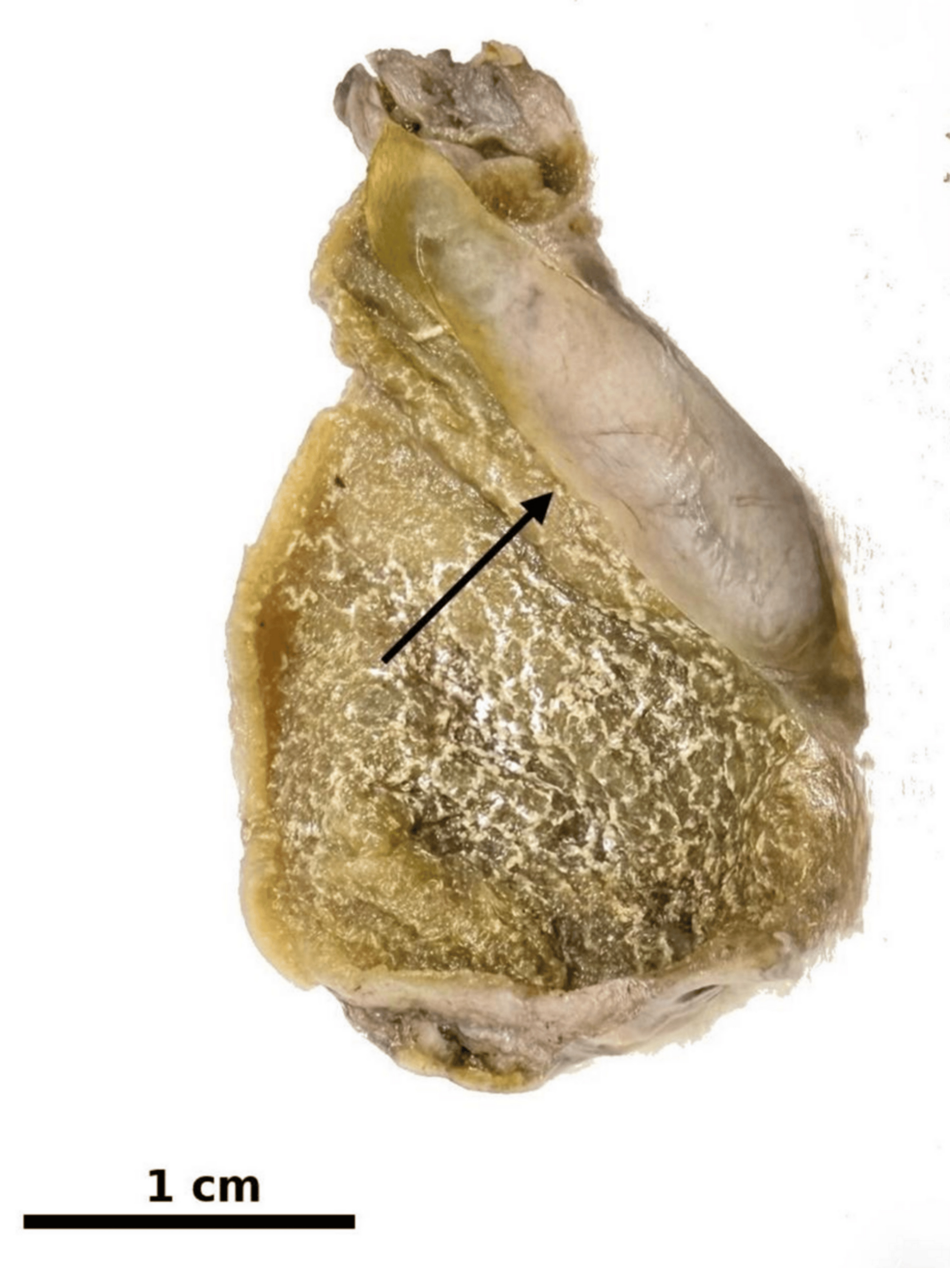
Gross examination of the gallbladder showing a cystic lesion (arrow) arising from the gallbladder wall. The surrounding mucosa demonstrates diffuse yellow stippling consistent with cholesterolosis. Scale bar = 1 cm.

Gross examination of the cholecystectomy specimen revealed an intact gallbladder measuring 5.9 × 3.5 × 1.9 cm. A subserosal semi-translucent cystic lesion measuring 3.5 × 1.5 × 1.4 cm was identified within the gallbladder wall, located approximately 1.0 cm from the cystic duct margin (Figure 2). On sectioning, the cyst was filled with clear fluid and had a smooth, glistening inner surface without papillary excrescences.

**Figure 3:**
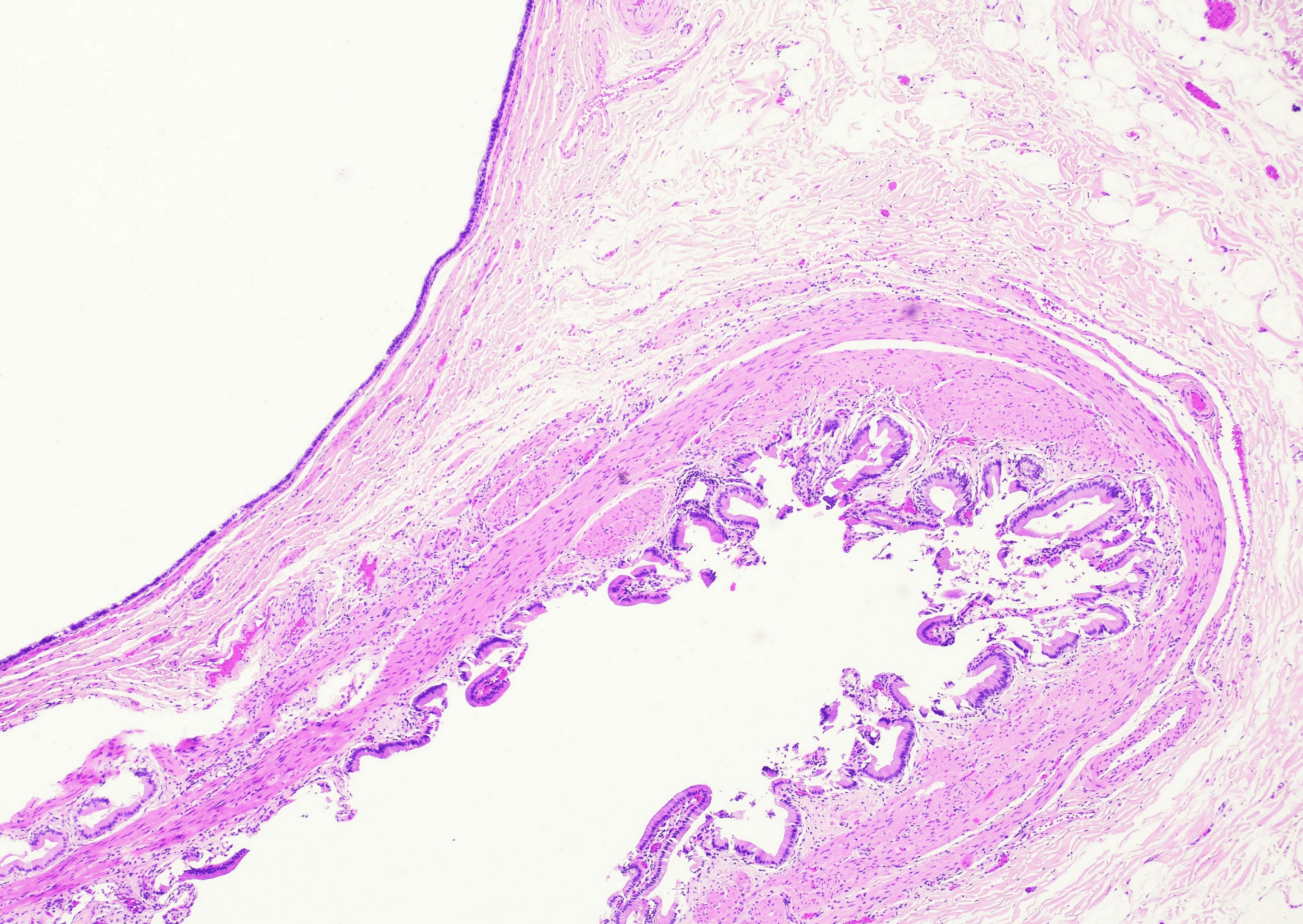
Low-power photomicrograph demonstrating a subserosal cyst within the gallbladder wall lined by folded epithelium, consistent with a ciliated foregut cyst (Hematoxylin and Eosin stain, original magnification 40x)

**Figure 4:**
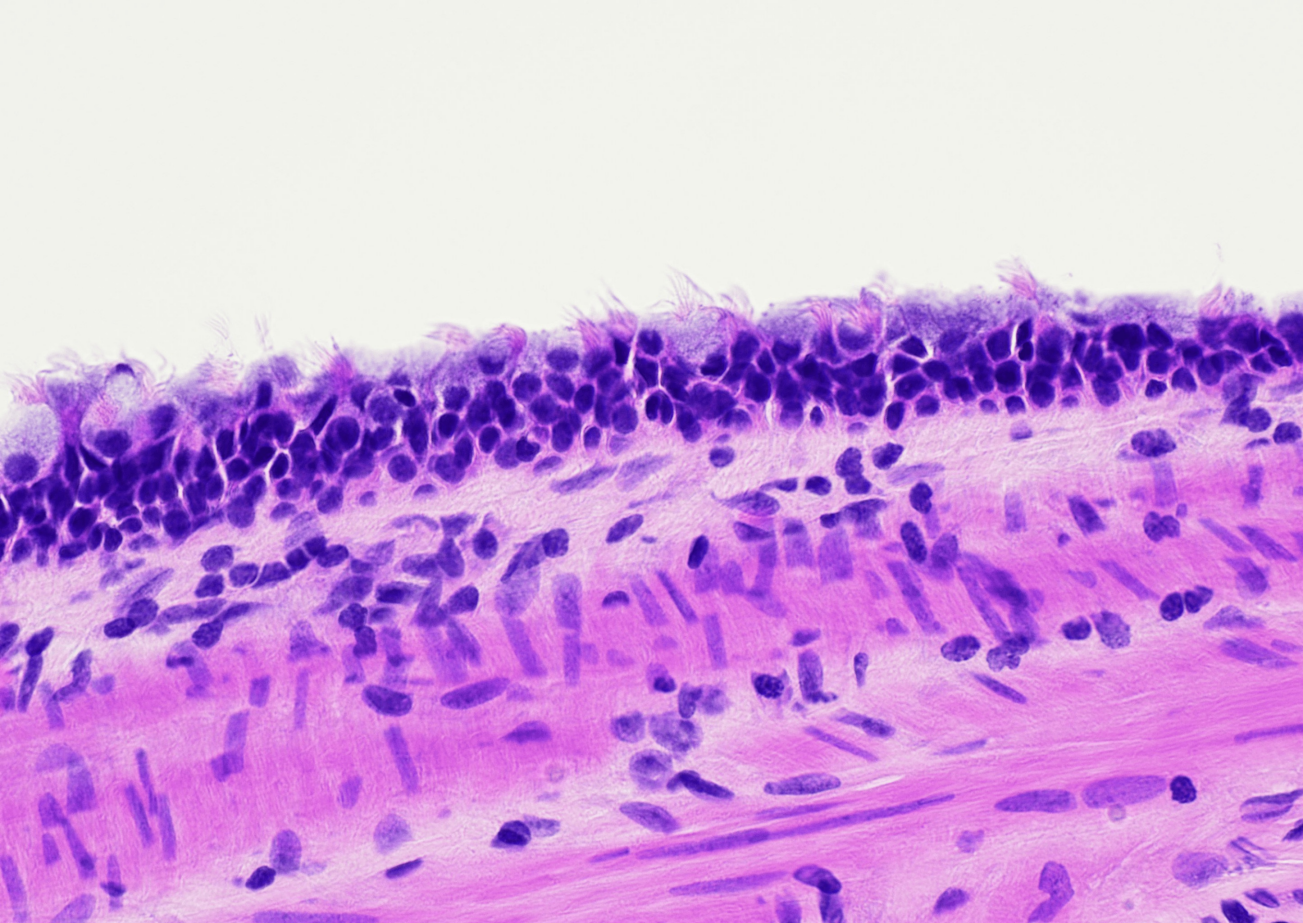
High-power photomicrograph demonstrating pseudostratified ciliated columnar (respiratory-type) epithelium with prominent apical cilia lining the cyst, consistent with a ciliated foregut cyst (Hematoxylin and Eosin stain, original magnification 400x)

Microscopic examination demonstrated a cyst located within the gallbladder wall lined by pseudostratified ciliated columnar (respiratory-type) epithelium with mucinous differentiation. At low magnification, the cyst was seen arising within the gallbladder wall (Figure 3). High-power examination demonstrated pseudostratified ciliated respiratory-type epithelium lining the cyst lumen (Figure 4). No epithelial atypia or malignancy was identified. These histologic findings were consistent with a ciliated foregut cyst of the gallbladder.

The patient’s postoperative course was uncomplicated. At follow-up, the patient was tolerating a regular diet, had normal bowel function, and his surgical incisions were healing well. He reported no recurrent abdominal pain, jaundice, or other complications.

## Discussion

Ciliated foregut cysts are rare congenital lesions that arise from abnormal budding or sequestration of the primitive foregut during embryologic development. These cysts are most commonly described in the mediastinum and liver but are rarely identified in the gallbladder [1-3,7-10]. Histologically, they are characterized by a lining of pseudostratified ciliated columnar (respiratory-type) epithelium, often accompanied by mucin-producing cells and occasionally smooth muscle within the cyst wall [1-3,7-10].

Ciliated foregut cysts belong to a spectrum of foregut-derived cysts that also includes bronchogenic cysts and esophageal duplication cysts [7-10]. When these cysts occur in the thorax, particularly in the mediastinum or lung parenchyma, they are often referred to as bronchogenic cysts. However, when similar cysts occur below the diaphragm, particularly in the liver or gallbladder, they are typically termed ciliated foregut cysts [7-10]. Histologically, these lesions share similar features, including a lining of pseudostratified ciliated columnar epithelium and mucin-secreting cells, reflecting their origin from the primitive foregut [7-10].

Gallbladder diverticula are outpouchings of the gallbladder wall that are lined by native gallbladder mucosa and communicate with the gallbladder lumen. They may be congenital or acquired and are often associated with increased intraluminal pressure or chronic inflammation [3,5,6]. Radiologically, gallbladder diverticula may appear as cystic structures adjacent to the gallbladder, making distinction from other cystic lesions difficult [3,5,6]. In contrast, ciliated foregut cysts are true cysts lined by respiratory-type epithelium and do not communicate with the gallbladder lumen [1,3]. In the present case, the lesion was initially interpreted radiologically as a diverticulum, highlighting this important diagnostic pitfall.

Ciliated foregut cysts involving the gallbladder are extremely uncommon, with only a limited number of cases reported in the literature [1-6]. Most lesions are discovered incidentally during imaging or surgery performed for unrelated conditions; however, some patients present with nonspecific symptoms, such as right upper quadrant abdominal pain, nausea, or dyspepsia, as seen in the present case [4].

Radiologic identification of these lesions is challenging because imaging findings are often nonspecific and may mimic other cystic lesions of the gallbladder [3,5,6]. On ultrasound or CT imaging, these cysts typically appear as well-circumscribed cystic structures adjacent to or within the gallbladder wall and may be misinterpreted as gallbladder diverticula, duplication cysts, or other benign cystic lesions [3,5,6].

Definitive diagnosis relies on histopathologic evaluation. The presence of pseudostratified ciliated respiratory-type epithelium lining the cyst wall is the key diagnostic feature that distinguishes ciliated foregut cysts from other cystic lesions of the gallbladder [3,6-8].

The differential diagnosis for cystic lesions associated with the gallbladder includes gallbladder diverticulum, duplication cyst, choledochal cyst, mucinous cystic neoplasm, and intracholecystic papillary neoplasm [3,5,6]. Gallbladder diverticula are lined by native gallbladder mucosa and communicate with the gallbladder lumen, whereas duplication cysts are typically lined by gastrointestinal-type epithelium [3,5]. Mucinous cystic neoplasms demonstrate ovarian-type stroma and mucinous epithelium [5,6]. In contrast, ciliated foregut cysts are lined by pseudostratified ciliated respiratory-type epithelium, which is the key diagnostic feature distinguishing this entity from other cystic lesions of the gallbladder [3,6-8].

Review of the literature demonstrates that most reported gallbladder ciliated foregut cysts measure less than 2-2.5 cm in greatest dimension [1-6]. The cyst in the present case measured 3.5 cm and represents one of the largest reported ciliated foregut cysts of the gallbladder in the literature. Table 1 summarizes previously reported cases of ciliated foregut cysts involving the gallbladder and their clinical characteristics.

**Table 1:** Reported cases of ciliated foregut cyst of the gallbladder in the literature

Author (Reference)	Year	Country	Journal	Age	Sex	Cyst Size	Clinical Presentation
Kakitsubata et al. [1]	1995	Japan	Abdominal Imaging	41	F	1.5 cm	Incidental finding
Muraoka et al. [2]	2003	Japan	Surgery Today	42	F	2.0 cm	Right upper quadrant pain
Hwang et al. [3]	2016	South Korea	Korean J Hepatobiliary Pancreat Surg	39	F	1.8 cm	Abdominal pain
Farrugia et al. [4]	2017	UK	BMJ Case Reports	34	M	2.5 cm	Symptomatic cyst
Bulut et al. [5]	2010	Turkey	Pathology Research International	41	F	1.2 cm	Right upper quadrant pain
Giakoustidis et al. [6]	2014	UK	J Gastrointestinal Liver Diseases	45	M	2.2 cm	Incidental finding
Present case	2026	USA	Current report	29	M	3.5 cm	Right upper quadrant pain, suspected gallbladder diverticulum

Although these cysts are generally considered benign, surgical excision is usually performed because the diagnosis is rarely established preoperatively, and imaging findings may mimic other gallbladder pathologies [3,5,6]. Recognition of this rare congenital lesion is important for pathologists and clinicians when evaluating cystic structures associated with the gallbladder.

Learning points

Ciliated foregut cysts of the gallbladder are extremely rare congenital lesions derived from primitive foregut remnants. These lesions may mimic gallbladder diverticula or other cystic abnormalities on imaging. Definitive diagnosis relies on histopathologic identification of pseudostratified ciliated respiratory-type epithelium. The cyst in the present case measured 3.5 cm, representing one of the largest reported ciliated foregut cysts of the gallbladder in the literature.

## Conclusions

Ciliated foregut cysts of the gallbladder are rare congenital lesions that may mimic gallbladder diverticula or other cystic lesions on imaging, making preoperative diagnosis challenging. Recognition of this entity and its characteristic histologic features is essential for accurate diagnosis. The cyst in the present case measured 3.5 cm and represents one of the largest reported ciliated foregut cysts of the gallbladder in the literature. This case highlights the importance of including ciliated foregut cyst in the differential diagnosis of cystic lesions associated with the gallbladder to ensure accurate diagnosis and appropriate management.
